# Coupling of ssRNA cleavage with DNase activity in type III-A CRISPR-Csm revealed by cryo-EM and biochemistry

**DOI:** 10.1038/s41422-019-0151-x

**Published:** 2019-02-27

**Authors:** Minghui Guo, Kaiming Zhang, Yuwei Zhu, Grigore D. Pintilie, Xiaoyu Guan, Shanshan Li, Michael F. Schmid, Zhuo Ma, Wah Chiu, Zhiwei Huang

**Affiliations:** 1 HIT Center for Life Sciences, School of Life Science and Technology, Harbin Institute of Technology, Harbin, Heilongjiang 150080 China; 2 Departments of Bioengineering, and of Microbiology and Immunology, and James H. Clark Center, Stanford University, Stanford, CA 94305 USA; 3 CryoEM and Bioimaging Division, SSRL, SLAC National Accelerator Laboratory, Stanford University, Menlo Park, CA 94025 USA

**Keywords:** Cryoelectron microscopy, Molecular biology

## Abstract

The type III CRISPR-Cas (clustered regularly interspaced short palindromic repeats-CRISPR-associated genes) systems are bacterially encoded adaptive immune systems for defense against invading nucleic acids. They accomplish this task through the coordinated cleavage of invading substrates of single-stranded RNA and DNA (ssDNA and ssRNA) by the Csm (type III-A) or Cmr (type III-B) effector complexes. The ssRNA is complementarily bound to the CRISPR RNA (crRNA). However, the structural basis for the DNase and RNase activation of the Csm nucleoprotein complex is largely unknown. Here we report cryo-EM structures of the Csm-crRNA complex, with or without target ssRNA, at near-atomic resolution. Our cryo-EM maps allow us to build atomic models of the key macromolecular components, including Cas10, Csm2, Csm3, Csm4, crRNA and the invading ssRNA. Our structure resolves unambiguously the stoichiometry and tertiary structures of the Csm protein complex and the interactions between protein components and the crRNA/ssRNA. Interestingly, the new atomic structures of the Csm proteins presented here are similar to those of previously known Csm proteins in other species despite their low sequence similarity. Our combined structural and biochemical data suggest that ssRNA cleavage is preferentially carried out near its 5’-end, that the extent of interactions among the ssRNA, crRNA and the protein components regulates the DNase activity of the Csm complex, and that the 3’ flanking sequence of ssRNA activates the Cas10 DNase activity allosterically.

## Introduction

Adaptive immune systems consisting of CRISPR (clustered regularly interspaced short palindromic repeats) and CRISPR-associated (Cas) genes defend prokaryotes from foreign pathogens. In a CRISPR-Cas system, Cas proteins form a complex with a small CRISPR RNA (crRNA) to detect and cleave invading nucleic acid (DNA or RNA) that has a complementary sequence to the crRNA.^1^ CRISPR-Cas systems fall into 2 classes, multi-Cas types I, III and IV (Class 1), and single-Cas types II, V and VI (Class 2). Both Csm and Cmr complexes in Class 1 can degrade ssDNA and ssRNA. The type III-A CRISPR-Csm complex contains five Cas proteins (Cas10, and Csm2–5) and a crRNA, with the predicted stoichiometry of Cas10_1_:Csm2_3_:Csm3_5_:Csm4_1_:Csm5_1_:crRNA_1_ (containing a 40 nt crRNA) for Csm-40, and Cas10_1_:Csm2_6_:Csm3_10_:Csm4_1_:Csm5_1_:crRNA_1_ (containing a 72 nt crRNA) for Csm-72, respectively.^2^ The Csm complex, with the RNA cleavage site located in Csm3 subunits, targets and cleaves ssRNA at 6-nucleotide intervals.^3^

To understand the molecular mechanisms of CRISPR-Cas-induced DNA/RNA degradation, great efforts have been made to resolve the structures of CRISPR-Cas systems. In 2015, the ~4.4 Å resolution structures of Cmr complex (from *Thermus thermophilus*) were obtained using cryo-electron microscopy (cryo-EM),^4^ and a 2.1 Å crystal structure of a chimeric Cmr complex (containing *Pyrococcus furiosus* Cmr2_1_-Cmr3_1_ and *Archaeoglobus fulgidus* Cmr4_3_-Cmr5_2_-Cmr6_1_) was also determined.^5^ These structures, displaying a spiral architecture, reveal the subunit stoichiometry and crRNA-binding sites, as well as a cleavage mechanism of the target ssRNA by the Cmr complex. For the Csm complex, EM structures have been obtained only at ~30 Å (*S. solfataricus*)^6^ and 17 Å (*Thermus thermophilus*),^7^ respectively. It is difficult to unambiguously deduce the stoichiometry and quaternary structure of the Csm complex from these low-resolution structures.

In this study, we determined the cryo-EM structures of the Csm complex from *Streptococcus thermophilus*, with or without target ssRNA, at 3.3–3.5 Å. Each of the two complexes can be separated computationally into two groups having different copy numbers of subunits Csm2 and Csm3. Each of the resulting maps was resolved well enough to allow us to build independent de novo models for the proteins, including a single copy of Cas10 and Csm4, variable copy numbers of Csm2 and Csm3, as well as the bound crRNA and target ssRNA. These models show the exact composition and structure of the Csm complex and offer the first atomic view of the Csm complex that suggests an allosteric mechanism of DNase activation regulated by ssRNA. Further mutagenesis experiments guided by our structures revealed a coupling mechanism between target ssRNA degradation by Csm3 and non-target DNA cleavage by Cas10.

## Results

### Overall near-atomic resolution cryo-EM structure of Type III-A CRISPR-Cas complex

**Fig. 1:**
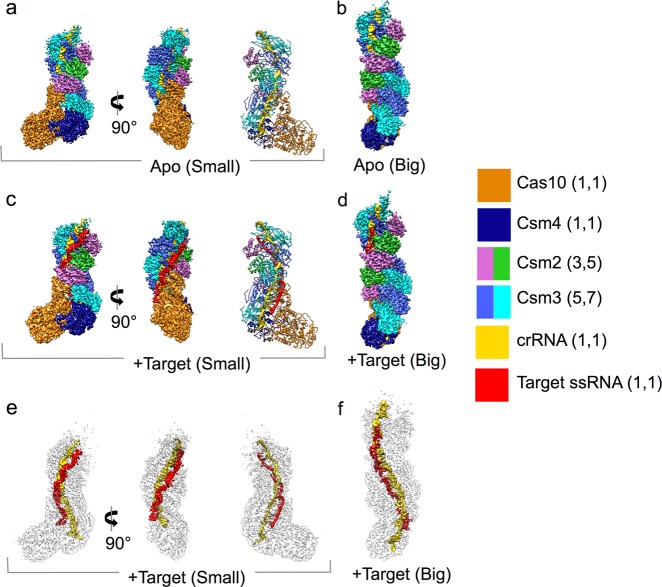
Overall structures of Type III-A CRISPR-Csm complex. **a** Apo small conformation. **b** Apo big conformation. **c** Target ssRNA-bound small conformation. **d** Target ssRNA-bound big conformation. In **a**, **c**, the surface representations for each component are shown in the middle and left panels; the map-derived models are shown in the right panel. **e**, **f** Models of crRNA and target ssRNA are highlighted within surface representations of the transparent maps. The legend on the right annotates the color of each subunit and the total number of subunits (in parenthesis) for the small and big states

We determined the near-atomic resolution structures of Csm proteins (Supplementary information, Fig. S1a) complexed with a 40 nt crRNA (from *Streptococcus thermophilus*), with or without 49 nt target ssRNA-bound, using cryo-EM single particle analysis. It should be mentioned that the D33 residues in Csm3 subunits of the target ssRNA-bound state were mutated (D33A) to eliminate ssRNA cleavage. The cryo-EM micrographs and 2D class averages of both complexes display clearly a worm-like shape with mixed particle sizes (Supplementary information, Figs. S1 and S2). These heterogeneous particle images were classified computationally into two particle populations each yielding three-dimensional (3-D) reconstructions for the apo state (big [3.4 Å], and small [3.3 Å]) (Fig. 1a, b; Supplementary information, Fig. S3) and for the target ssRNA-bound state (big [3.4 Å], and small [3.5 Å]) (Fig. 1c–f; Supplementary information, Fig. S4 and Movie S1). All four maps have an elongated shape, with two spirally stacked columns terminating in a foot-like shape at the bottom. One column consists of multiple repeating Csm3 subunits and a single Csm4, whereas the other contains multiple repeating Csm2 subunits and a single Cas10.

**Fig. 2:**
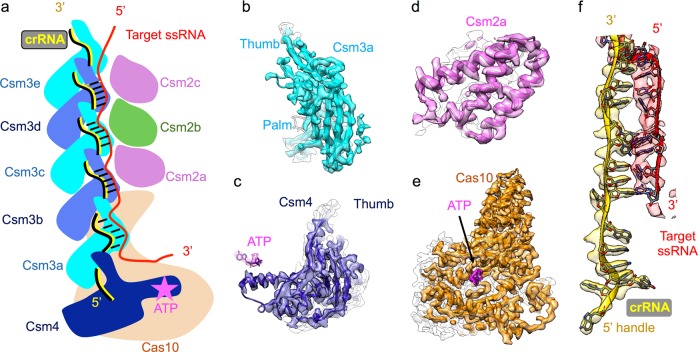
Structures of individual subunits. **a** The cartoon shows the architecture of the Csm complex with target ssRNA bound using small conformation as an example. **b-f** Individual densities and models of Csm3, Csm4, Csm2, Cas10, crRNA, and target ssRNA

In both states (with or without target ssRNA), the only difference between the respective big and small maps was the number of repeating Csm2 and Csm3 subunits. In total, the big maps contain 7 repeating Csm3 and 5 repeating Csm2 subunits, while the small maps contain 5 copies of Csm3 and 3 copies of Csm2 (Figs.1 and 2a). In other words, the extra length in the big maps is attributable to the 2 more copies of Csm2 and Csm3 proteins. We will mainly discuss the structures of the small complex in this paper as the big structures do not contribute to further mechanistic understanding of the functional activities.

### Detailed structures of individual subunits

The Csm3 proteins in the complex have the same palm-thumb shaped conformation (Fig. 2b; Supplementary information, Movie S1) as previously observed in type I CRISPR-Cas complexes.^8,9^ Csm4 also displays a thumb-like protrusion and has a helix extending to a nearby ATP molecule bound to Cas10 (Fig. 2c; Supplementary information, Movie S1). Csm2 is composed of 6 alpha helices and adopts a small globular shape (Fig. 2d; Supplementary information, Movie S1). Cas10 is the largest subunit in the Csm complex and contains an ATP-binding pocket (Fig. 2e; Supplementary information, Movie S2).

The quality of the maps is sufficient to allow us to build de novo models guided by well-connected backbone densities including the beta strands and loops, as well as many visible amino acid side chains (Fig. 2b–e) and nucleotide base densities in the crRNA (Fig. 2f). The quality of the models is confirmed by a number of quantitative measures including the MolProbity statistics reflective of model stereochemistry^10^ (Supplementary information, Table S1), side chain Z-scores reflective of their resolvability^11^ (Supplementary information, Fig. S5), per-residue cross-correlation coefficients reflective of map and model agreement, and atomic displacement parameters reflective of model accuracy^12^ (Supplementary information, Fig. S6). The target ssRNA is slightly less resolved but still well enough for us to trace the backbone connectivity of the core region with an apparent base-pairing to the crRNA (Fig. 2f).

In our model, the palms of Csm3 associate with each other along the spiral architecture. In addition, the thumb of each Csm3 holds the crRNA against the palm of the next Csm3 protein (Fig. 2a; Supplementary information, Fig. S7 and Movie S3). The Csm4 also contains a thumb-like shape which holds the crRNA against the palm of Csm3a (Fig. 2a). In the other spiral column, the Csm2 proteins stack on the top of Cas10; the loops in each Csm3 containing the residue (Asp33) implicated in cleaving the target ssRNA are stabilized by its adjacent Csm2 subunits (Supplementary information, Fig. S7).

### Allosteric activation of the DNase activity by the 3’ flanking sequence of ssRNA

**Fig. 3:**
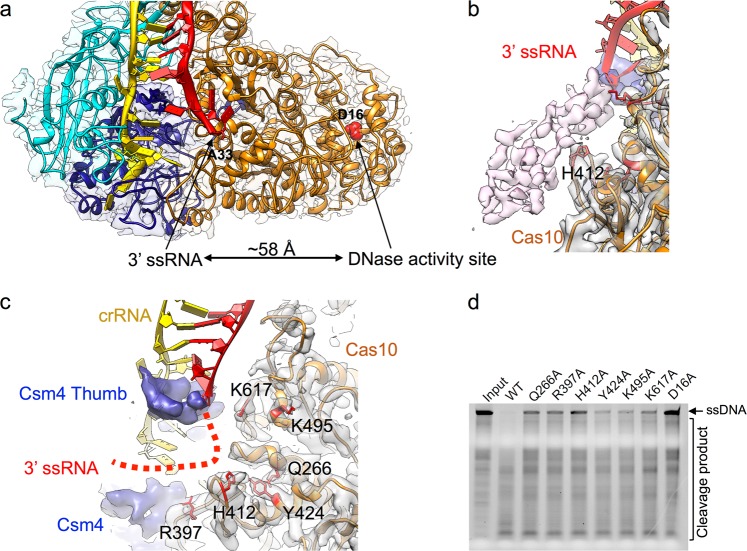
Allosteric activation of DNase activity by the 3’ flanking sequence of ssRNA. **a** The last visible 3’-terminal base (A33) of ssRNA is ~58 Å away from the DNase activity site (residue D16, red sphere atoms). **b** The potential pathway of the 3’-flanking sequence of ssRNA showed by lower-threshold density (light purple). **c** The interface between 3’ flanking sequence of ssRNA and Cas10. **d** Mutations of residues at the interface between Cas10 and the 3’ flanking sequence of ssRNA impaired its DNase activity

Since data have shown that the 3’ flanking sequence of ssRNA plays a critical role in initiating Cas10’s DNase activity,^13^ we next sought to elucidate how Cas10’s DNase activity is activated by the 3’ flanking segment of ssRNA. Our structure clearly shows that the DNase active site in Cas10 is located on the opposite side from the last modeled 3’-base of the ssRNA (Fig. 3a). Although the model of the 3’ flanking sequence of the ssRNA cannot be built due to its high flexibility, lowering the threshold reveals a possible locality of the 3’ flanking sequence in the map of the target ssRNA-bound state (Fig. 3b; Supplementary information, Movie S4), which does not occur in the map of the apo state. Since these bases are far away from the DNase active site (Fig. 3a), the influence of the ssRNA binding to the Csm complex must be accomplished through an allosteric mechanism. Previous data showed that DNase activation triggered by the 3’ flanking region of ssRNA is independent of its specific sequence.^2^ Therefore, the allosteric activation of the DNase activity may be accomplished through the interaction between the enzyme and the sugar-phosphate backbone of the 3’ flanking sequence of ssRNA. Our biochemical data showed that mutations of Q266, R397, H412, Y424, K495 and K617 in Cas10 largely impaired DNase activity, supporting that these residues play a critical role for allosterically activation of DNase activity (Fig. 3c, d).

### DNase activity is regulated by ssRNA cleavage

**Fig. 4:**
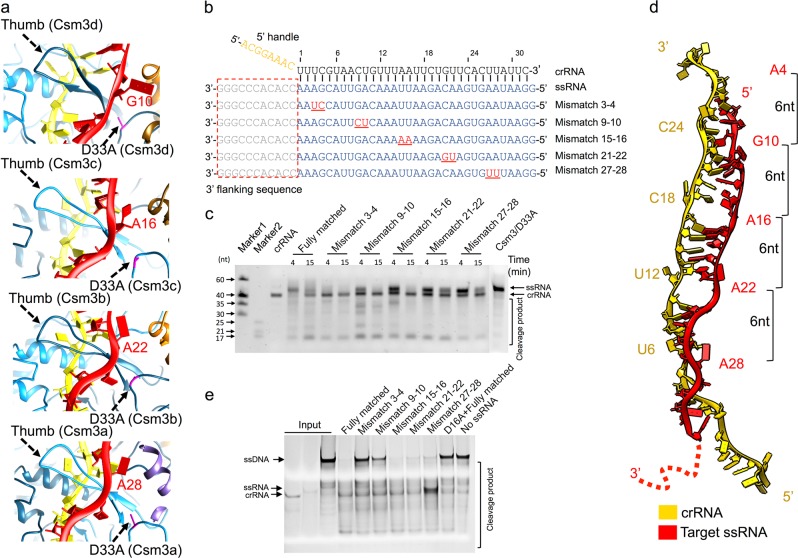
The target ssRNA and ssDNA cleavage mechanism of the Csm complex. **a** Zoom-in views of Csm3 thumb regions intercalating the crRNA and target ssRNA. **b** Sequences of crRNA and ssRNA. The mismatches between ssRNA and crRNA are underlined in red. The numerology of the mismatched nucleotides is based on the crRNA. **c** In vitro ssRNA cleavage assay using 2-base pair mismatched crRNA. **d** Model of crRNA and target ssRNA nucleotides. Target ssRNA and crRNA nucleotides which are not base-paired due to Csm4 and Csm3 thumbs are shown in 1-letter code and specifier. **e** In vitro ssDNA cleavage assay using 2-base pair mismatched crRNA and non-target ssDNA

Next, we sought to determine the coupling mechanism between RNase and DNase activities. The heteroduplex of crRNA and target ssRNA show a repeating base pairing pattern along the spiral configurations of Csm3 and Csm2 subunits. The pattern consists of 5 base pairs, followed by a base pair disrupted by the thumb of a Csm3 or Csm4 protein (Figs. 2a, 4a; Supplementary information, Fig. S7a and Movie S3). Therefore, the Csm3 or Csm4 thumb intercalates within the crRNA-ssRNA duplex at 6-nucleotide intervals. To examine whether the ssRNA is cleaved sequentially, randomly, repeatedly, or specifically, we performed in vitro cleavage experiments using a series of synthesized target ssRNA with different mismatches (3’-5’) to the crRNA (Fig. 4b). In describing our results, we use the nucleotide mismatch of ssRNA with reference to the numbering from 5’ to 3’ of the crRNA. Mismatches in the ssRNA closer to its 5’-end result in lower ssRNA cleavage activities compared to the mismatches in the 3’-end (Fig. 4c). For instance, ssRNA mismatch 21–22 and 27–28 were cleaved at much lower levels compared with ssRNA mismatch 3–4, suggesting that the ssRNA is likely to be cleaved specifically, with the preferred cleavage sites of Csm3 locating nearer to the ssRNA 5’-end. To test this hypothesis, we used a 5’-labeled ssRNA in the in vitro cleavage experiment. The 10-nt RNA product was the dominant cleavage product since the cleavage started, indicating that the cleavage preferentially happens at nucleic acid 24 of ssRNA (Fig. 4d; Supplementary information, Fig. S8a and b). Moreover, our cleavage assay using 2’-deoxy-substituted ssRNAs at nucleic acids 6, 12, 18, 24, and 30 also reveals that the cleavage activity is largely abrogated by the ssRNA carrying this mutation at 24 (Supplementary information, Fig. S8c and d), further supporting the notion that the cleavage at 5’-ssRNA-proximal site plays a critical role for the subsequent 3’-ssRNA degradation. Notably, no 6, 12, 18, or 24-nt RNA cleavage products were found (Supplementary information, Fig. S8c and d), either in the wild-type or any of these mismatch mutant groups, as they would be if the ssRNA were cleaved at more than one of its 6-nt-spaced possible cleavage sites. This suggests that the ssRNA is cleaved only once, and is then dissociated from the complex rather promptly. These results rationalize that the Csm3 cleaves the target ssRNA molecule one time; usually, but not always, at a preferential site or sites near its 5’-proximal end. This leads to the decreased binding affinity of ssRNA with crRNA, the subsequent dissociation and release of ssRNA products from the Csm complex (Fig. 4d), and the cessation of ssDNA cleavage activity. Accordingly, the cleavage activities for both the non-target and target ssDNA with mismatched ssRNA substrates display a reversed pattern compared with the ssRNA cleavage result (Fig. 4e; Supplementary information, Fig. S8e). The mismatches in ssRNA 5’-end (e.g., 21–22, 27–28) resulted in much stronger DNA cleavage activities compared with the mismatches in its 3’-end (e.g., 3–4). This is because the reduced ssRNA cleavage at these preferred sites allows a longer persistence of the DNase activity. Moreover, ssRNA mismatch 1–2 further impaired DNA cleavage activity compared with mismatch 3–4 (Supplementary information, Fig. S8f), possibly resulting from the destabilization of the 3’ flanking-proximal region of ssRNA, thereby inhibiting the 3’ flanking region from interacting with the binding site of Cas10 for allosteric activation. These findings further support the idea that a stable interaction between the 3’ flanking-proximal region of ssRNA and the Csm complex is necessary for DNase activity. Taken together, our data suggest that DNase activity is coupled to and modulated by the cleavage of ssRNA upon ssRNA binding to the Csm complex.

### ATP binding site in Cas10

**Fig. 5:**
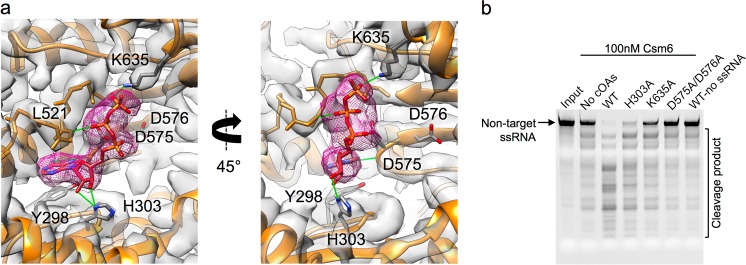
ATP binding residues in Cas10 are crucial for the activity of Csm6. **a** The residues Y298, H303, D575, D576, K635, and L521 from Cas10 (orange ribbon), surrounding the resolved ATP molecule (purple mesh surface), are shown. Distances (green sticks) between the ATP molecule and these residues vary from 3.0 Å to 3.2 Å. **b** Residue mutations within the ATP-binding pocket of Cas10 impair the non-specific RNase activity of Csm6

Previous study showed that target ssRNA binds to the Csm effector complex thereby triggering the ATP-dependent synthesis of cyclic oligoadenylates (cOAs) by Cas10. cOAs bind Csm6 nuclease to activate its non-specific ssRNA degradation activity.^14^ In our structure, an endogenous ATP molecule was observed in both the apo and target ssRNA-bound states, within the Cas10-formed binding pocket (Supplementary information, Movie S2). The adenine ring of the ATP stacks against the aromatic side chain of Tyr298 in Cas10 (Fig. 5a). The pentose moiety and phosphoryl atoms of the ATP molecule are in close proximity to residues His303, Lys635, Asp575/576, and Leu521 of Cas10 (Fig. 5a). For example, the N atoms of His303 and Lys635 are located ~3.1 Å away from the phosphoryl oxygens in the ATP, and the Asp575 residue is also close to the phosphoryl atoms with the distance of ~2.9 Å. Mutation of His303, Lys635 or Asp575/576 in Cas10 severely impaired Csm6 RNase activity that degrades the non-specific ssRNA (Fig. 5b), consistent with a previous study showing that ATP is required for Cas10 to activate Csm6.^14^

## Discussion

In this study, we obtained two sets of 3D reconstructions of the Csm complex at near-atomic resolutions. The first set is the Csm complex with crRNA, a 3.4-Å big Csm complex, and a 3.3-Å small Csm complex. We also obtained two other maps of Csm complexes with target ssRNA at similar resolutions. The results showed that the Csm complex displays a spiral-like architecture, with two spirally stacked density columns, including a single Cas10, a single Csm4, and multiple copies of Csm2 and Csm3. The number of Csm2 and Csm3 subunits differentiate the big Csm complex from the small one. De novo models can be built for all of our maps. The Csm proteins in our models are structurally similar to previously known Csm proteins in other species, although they have low sequence similarity (Supplementary information, Fig. S9 and Movie S5). In our structure, the least resolved component is Csm5 at one end of the complex, near the 3’-end of the crRNA, implying a flexible conformation of this subunit.

The structural comparison between the apo and target ssRNA-bound states of the Csm complex revealed no substantial structural difference between the two structures. However, we do notice a small shift in the Csm2 proteins in the target ssRNA-bound state compared to the apo state (Supplementary information, Fig. S7c). The shift is similar to the ‘rotation’ noted in the Cmr complex^13^ and may indicate that Csm2 is slightly affected by or may have a role in the binding of the target ssRNA. The shift is more pronounced in Csm2a (~1.2 Å) than in Csm2b (~0.9 Å) or Csm2c (negligible) (Supplementary information, Fig. S7c). However, it is unclear whether the Csm2 shift is coupled to the ssRNA degradation. The apo and target ssRNA-bound states also correspond to DNase inactive and activated states of the complex, respectively. We found that DNase activity is coupled to and regulated by the ssRNA cleavage upon ssRNA binding to the Csm complex. In other words, DNase activity is activated when the invading ssRNA binds to the Csm complex and is inactivated when the ssRNA has been degraded, preventing the constitutive DNase activity that might be harmful to the bacterium. Previous data also showed that target ssRNA binding activates DNase activity, that subsequent ssRNA cleavage represses DNA degradation, and that the 3’ flanking sequence is required for the activation of DNase activity.^2,13^ Our structure and biochemical data suggest a mechanistic model for how these observations are linked together. The ssRNA degradation initiated at the preferred cleavage site near its 5’-proximal end would allow the Csm complex to remain in the intact 3’ flanking ssRNA-bound state for a certain time window to activate the DNase activity. These coordinated events explain how the Csm complex achieves its functions to cleave the invading ssRNA effectively and to activate the DNAase for a limited time.

## Materials and methods

### Protein expression and purification

Genomic DNA was obtained from *Streptococcus thermophilus* (St) DGCC8004 strain. Point mutations in StCsm gene were introduced by Fast Site-Directed Mutagenesis Kit and verified by DNA sequencing. StCsm complexes were obtained as described previously.^2^ StCsm gene covering *cas6-cas10-csm2-csm3-csm4-csm5-csm6-csm6’* gene cassette was cloned into the pCDFDuet-1 expression vector via *Bgl*II and *Avr*II restriction sites to generate plasmid pCas/Csm. CRISPR locus containing five 36-nt length repeats interspaced by four identical 36-nt spacers S3 of the *S. thermophilus* DGCC8004 CRISPR2 system was obtained and cloned into the pACYC-Duet-1 vector to generate a plasmid pCRISPR_S3. Individual Csm2 gene was cloned into pET-28a_N_His. All three plasmids were co-expressed in *Escherichia coli* C43 (DE3) grown at 37 °C in LB medium supplemented with streptomycin (25 μg/μL), kanamycin (25 μg/μL), and chloramphenicol (17 μg/μL). Expression of the StCsm-complex was induced by 0.3 mM isopropyl β-D-1-thiogalactopyranoside (IPTG) at 20 °C. After overnight induction, the cells were collected by centrifugation and resuspended in buffer A (25 mM Tris-HCl, pH 8.0, 150 mM NaCl, 15 mM imidazole) supplemented with 1 mM protease-inhibitor PMSF (phenylmethylsulfonyl fluoride, Sigma). The cells were subjected to lysis by sonication and cell debris was removed by centrifugation at 23,708 × *g* for 40 min at 4 °C. The lysate was first purified using Ni^2+^-NTA resin. The beads were washed and the bound proteins were eluted by buffer B (25 mM Tris-HCl, pH 8.0, 100 mM NaCl, 250 mM imidazole) for 1 h at 4 °C. Further fractionated by heparin sepharose column and ion exchange chromatography via FPLC (AKTA Pure, GE Healthcare), StCsm-crRNA binary complex was applied onto size-exclusion chromatography (Superdex 200 increase 10/300 GL, GE Healthcare) with buffer C (10 mM Tris-HCl, pH 8.0, 150 mM NaCl, 3 mM DTT). Purified complexes were concentrated to 2–4 mg/mL, flash frozen in liquid nitrogen, and stored at −80 °C.

Csm6 genes were amplified separately by means of PCR, using genomic *S. thermophilus* DGCC8004 DNA as a template, and cloned into the pGEX-6P-1 vector. The cells were resuspended in buffer D (25 mM Tris-HCl, pH 8.0, 1 M NaCl, 3 mM DTT) supplemented with 1 mM protease-inhibitor PMSF (phenylmethylsulfonyl fluoride, Sigma). Then the cells were subjected to lysis by sonication and cell debris was removed by centrifugation at 23,708 × *g* for 40 min at 4 °C. The lysate was first purified using glutathione sepharose 4B (GS4B) beads (GE Healthcare). The beads were washed and the bound proteins were cleaved by the PreScission protease in buffer E (25 mM Tris-HCl, pH 8.0, 300 mM NaCl, 3 mM DTT) overnight at 4 °C to remove the GST tag. The cleaved protein was eluted from GS4B resin. Further fractionation by heparin sepharose column and size-exclusion chromatography via FPLC (AKTA Pure, GE Healthcare) were conducted.

### In vitro transcription and purification of ssRNA

The ssRNA was transcribed in vitro using T7 polymerase and purified using corresponding concentration denaturing polyacrylamide gel electrophoresis. Transcription template (dsDNA) for ssRNA was generated by PCR. Buffer containing 0.1 M HEPES-K pH 7.9, 12 mM MgCl_2_, 30 mM DTT, 2 mM Spermidine, 2 mM each NTP, 80 μg mL^−1^ home-made T7 polymerase, and 400 nM transcription template was used for the transcription reactions. The reactions were processed at 37 °C for 5 h and stopped by 1 h at −80 °C. Pyrophosphate was precipitated with Mg^2+^ at 4 °C, and DNA templates were precipitated with Spermidine. After the precipitation was removed, RNAs were precipitated by ethanol precipitation. The RNA-containing pellets were then resuspended and purified by gel electrophoresis on a denaturing (8 M urea) polyacrylamide gel. RNA bands were excised from the gel and recovered with Elutrap System, followed by ethanol precipitation. RNAs were resuspended in diethyl pyrocarbonate H_2_O and stored at −80 °C. Nucleic acid sequences used in this study are listed in Supplementary information, Table S2.

### Reconstitution of the StCsm-crRNA-ssRNA ternary complex

To assemble the StCsm-crRNA-ssRNA complex, StCsm-crRNA complex was incubated with ssRNA at a molar ratio of 1:2 at room temperature for 5 min and 4 °C for 1 h. The complex was applied onto size-exclusion chromatography (Superdex 200 increase 10/300 GL, GE Healthcare) with buffer C (10 mM Tris-HCl, pH 8.0, 150 mM NaCl, 3 mM DTT) to remove excess ssRNA. The purity of the protein was monitored at all stages of the purification process using SDS-PAGE (polyacrylamide gel electrophoresis) and visualized by Coomassie blue staining. SsRNA and crRNA were monitored using 10% TBE-Urea denaturing gel and visualized by ethidium bromide staining. Purified complexes were concentrated to 2–4 mg/mL, flash frozen in liquid nitrogen, and stored at −80 °C.

### Endonuclease cleavage activity assays

In vitro DNA cleavage reactions were performed in the 15 μL reaction buffer (33 mM Tris-acetate [pH 7.6], 66 mM K-acetate, 0.1 mg mL^−1^ BSA, 1 mM EDTA) containing 700 nM StCsm-crRNA, 2 μM ssRNA, and 2.7 μM ssDNA at 37 °C for 30 min. Reactions were initiated by the addition of 5 mM MnCl_2_ and stopped by adding 2 × TBE-urea loading buffer and quenching at 95 °C for 5 min. Cleavage products were run on 10% TBE-Urea denaturing gel at room temperature in 1 × TBE running buffer and visualized by EB staining.

In vitro RNA cleavage reactions were performed in the 10 μL cleavage buffer (25 mM Tris-HCl [pH 8.0], 2 mM MgCl_2_, 60 mM NaCl) containing 400 nM StCsm-crRNA, 4 μM ssRNA at 37 °C. The samples were collected at timed intervals and 10 μL of the reaction mixture was mixed with 2 × TBE-urea gel loading buffer, followed by quenching at 95 °C for 5 min. Then the cleavage products were treated as described above.

For the cleavage assay of the 2’-deoxy-substituted RNAs, cleavage reactions were performed in the 10 μL cleavage buffer (25 mM Tris-HCl [pH 8.0], 2 mM MgCl_2_, 60 mM NaCl) containing 700 nM StCsm-crRNA and 4.8 μM ssRNA at 37 °C. The samples were collected at timed intervals and 10 μL of the reaction mixture was mixed with 2 × TBE-urea gel loading buffer. Then the cleavage products were treated as described above.

In vitro labeled ssRNA cleavage reactions were performed in a 10 μL system containing 70 nM StCsm-crRNA and 0.4 μM 5’-end labeled ssRNA. The 5’-end labeling was accomplished using the 5’-oligonucleotide kit (Vectorlabs) with a maleimide-IR800 probe (LI-COR Biosciences, Lincoln.NE). Cleavage reactions were conducted at 37 °C in the cleavage buffer (25 mM Tris-HCl [pH 8.0], 2 mM MgCl_2_, 60 mM NaCl) and the samples were taken at the indicated time points (0.5, 1, 3, 5, 10, and 20 min). Reactions were stopped by adding 2 × TBE-urea loading buffer and quenching at 95 °C for 5 min. Cleavage products were run on 10% TBE-Urea denaturing gel at room temperature in 1 × TBE running buffer and visualized by fluorescence imaging.

### StCsm-mediated synthesis of cyclic oligoadenylates (cOAs) for Csm6 nuclease assay

The synthesis reactions of cOAs by StCsm were performed in the 100 μL reaction buffer (33 mM Tris-acetate [pH 7.6], 66 mM K-acetate, 0.1 mg mL^−1^ BSA) containing 200 nM StCsm-crRNA, 200 nM ssRNA, 50 μM ATP at 37 °C for 1.5 h. Reactions were initiated by addition of 10 mM Co^2+^ and stopped by adding 15 mM EDTA. StCsm6 nuclease assays were conducted in the reaction buffer (33 mM Tris-acetate [pH 7.6], 66 mM K-acetate, 0.1 mg mL^−1^ BSA) containing 100 nM Csm6, 2 μL cOA synthesis reactions mix, 6.7 μM ssRNA at 37 °C for 10 min. Reactions were stopped by adding 2 × TBE-urea loading buffer and quenching at 95 °C for 5 min. Cleavage products were treated as described above.

### Cryo-electron microscopy data acquisition

Two samples of *Streptococcus thermophilus* CRISPR-Csm complex, apo state (with crRNA) and target ssRNA-bound state (with crRNA and target ssRNA), were diluted to a final concentration of ~0.4 mg/mL. Three microliters of samples were applied onto glow-discharged 200-mesh R2/1 Quantifoil grids. The grids were blotted for 4 s and rapidly cooled in liquid ethane using a Vitrobot Mark IV (FEI) with 4 °C and 100% humidity. The samples were screened using Talos Arctica cryo-electron microscope (FEI) operated at 200 kV and then imaged in a Titan Krios cryo-electron microscope (FEI) with GIF energy filter (Gatan) at a magnification of 130,000 × (corresponding to a calibrated sampling of 1.06 Å per pixel). Micrographs were recorded with a Gatan K2 Summit direct electron detector, where each image is composed of 30 individual frames with an exposure time of 6 s and a dose rate of 7 electrons per second per Å^2^. A total of 3782 movie stacks for apo state and 5123 movie stacks for the target ssRNA-bound state were collected with a defocus range of 0.9–3 μm.

### Single particle image processing and 3D reconstruction

All micrographs were motion-corrected using MotionCor2^15^ and CTF-corrected using CTFFIND4.^16^ All particles were autopicked using NeuralNet option in EMAN2^17^ and further checked manually, yielding 304,318 particles from selected 3600 micrographs for the apo state, and 390,850 particles from selected 4,950 micrographs for the target ssRNA-bound state. Then, particle coordinates were imported to RELION 2.1,^18^ where four rounds of 2D classification were performed to remove poor 2D class averages. Two datasets of 179,039 particles for apo state and 250,198 particles for the target ssRNA-bound state were used for Ab-initio 3D reconstruction in CryoSparc Version 0.65,^19^ and two conformations, “big” and “small”, of each state were obtained. Further heterogeneous refinement using the 2 maps as initial models were used to remove bad classes. Final 3D refinement was performed in CryoSPARC using 84,024 particles for apo big Csm complex, 81,540 particles for apo small Csm complex, 73,422 particles for target ssRNA-bound big Csm complex, and 50,092 particles for target ssRNA-bound small Csm complex, resulting in corresponding maps with resolutions of 3.4 Å, 3.3 Å, 3.4 Å, and 3.5 Å, respectively.

### Model building and optimization

Model building was first carried out based on the 3.4 Å reconstruction map of the apo Csm complex. The atomic coordinates of Csm1from *Thermococcus onnurineus* (PDB accession number: 4UW2), Csm2 from *Thermotoga maritima* (PDB accession number: 5AN6), Csm3 from *Methanopyrus kandleri* (PDB accession number: 4N0L), and Csm4 from *Methanocaldococcus jannaschii* (PDB accession number: 4QTS) were manually fitted into the density map of each protein component by CHIMERA^20^ to generate a starting model, followed by manual rebuilding using COOT.^21^ The refined models of each protein component were fitted into the density map of Csm complex (small) by CHIMERA. The crRNA and target ssRNA were built de novo using COOT. Secondary structure element prediction of each subunit was performed using the PSIPRED workbench.^22^ Sequence alignment was carried out using Clustal W.^23^ All models were refined using phenix.real_space_refine^24^ application with secondary structure element and geometry restraints. The final models were evaluated by MolProbity.^10^ Statistics of the map reconstruction and model optimization are presented in Supplementary information, Table S1. The agreement between map and model was assessed by the correlation coefficient per residue^12^ and the Z scores computed for each amino acid residues^11^ were used to quantify the resolvability of the side chain densities.

### Data availability

Cryo-EM structures and atomic models have been deposited to the Electron Microscopy Data Bank and the Protein Data Bank under accession codes EMD-0516, EMD-0517, EMD-0518, EMD-0519 and PDB ID 6NUD, and 6NUE.

## Supplementary information

Supplementary information, Figure S1

Supplementary information, Figure S2

Supplementary information, Figure S3

Supplementary information, Figure S4

Supplementary information, Figure S5

Supplementary information, Figure S6

Supplementary information, Figure S7

Supplementary information, Figure S8

Supplementary information, Figure S9

Supplementary information, Movie S1

Supplementary information, Movie S2

Supplementary information, Movie S3

Supplementary information, Movie S4

Supplementary information, Movie S5

Supplementary Movie Legends

Supplementary information, Table S1

Supplementary information, Table S2
