# Supplementary material for: Coupling of ssRNA cleavage with DNase activity in type III-A CRISPR-Csm revealed by cryo-EM and biochemistry
Source: Cell Res. 2019 Feb 27;29(4):305–12. doi: 10.1038/s41422-019-0151-x (PMC6461802; doi:10.1038/s41422-019-0151-x)
Supplement: Supplementary file 1 — Supplementary information, Figure S1 [file 41422_2019_151_MOESM1_ESM.pdf]

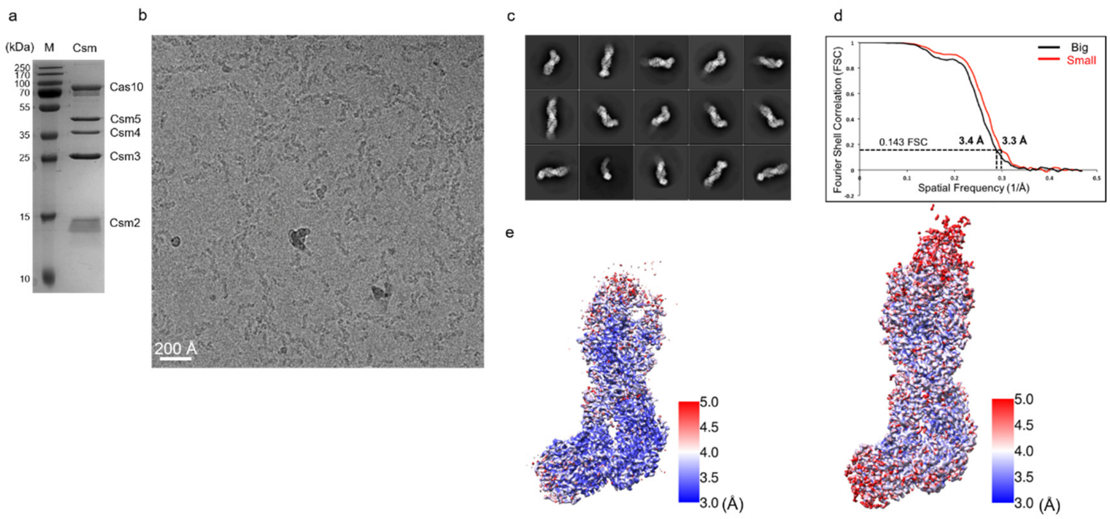

Fig. S1 Single particle 3D reconstructions of apo Csm complexes. **a** SDS-PAGE and Coomassie blue staining of the Csm complex from gel filtration. MM, molecular mass marker. **b** Representative field of the cryo-EM image. **c** Representative reference-free 2D class averages. **d** Gold standard FSC plot for the 3D reconstructions. **e** Resolution distribution (ResMap) for small (left) and big (right) complexes.
