# Supplementary material for: Coupling of ssRNA cleavage with DNase activity in type III-A CRISPR-Csm revealed by cryo-EM and biochemistry
Source: Cell Res. 2019 Feb 27;29(4):305–12. doi: 10.1038/s41422-019-0151-x (PMC6461802; doi:10.1038/s41422-019-0151-x)
Supplement: Supplementary file 2 — Supplementary information, Figure S2 [file 41422_2019_151_MOESM2_ESM.pdf]

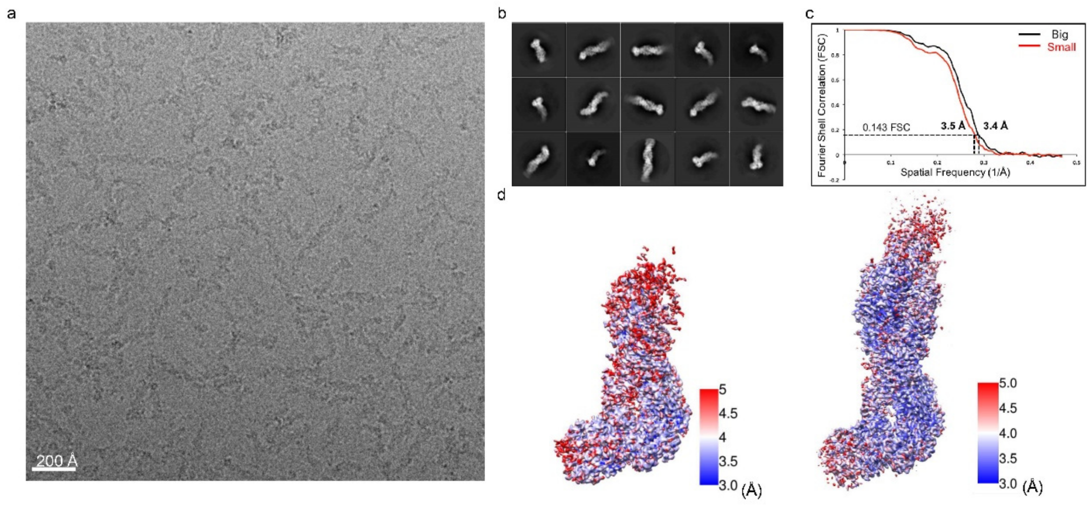

Fig. S2 Single particle 3D reconstructions of target ssRNA-bound Csm complexes. **a** Representative field of the cryo-EM image. **b** Representative reference-free 2D class averages. **c** Gold standard FSC plot for the 3D reconstructions. **d** Resolution distribution (ResMap) for small (left) and big (right) complexes.
