# Supplementary figures and images for: Coupling of ssRNA cleavage with DNase activity in type III-A CRISPR-Csm revealed by cryo-EM and biochemistry
Source: Cell Res. 2019 Feb 27;29(4):305–12. doi: 10.1038/s41422-019-0151-x (PMC6461802; doi:10.1038/s41422-019-0151-x)

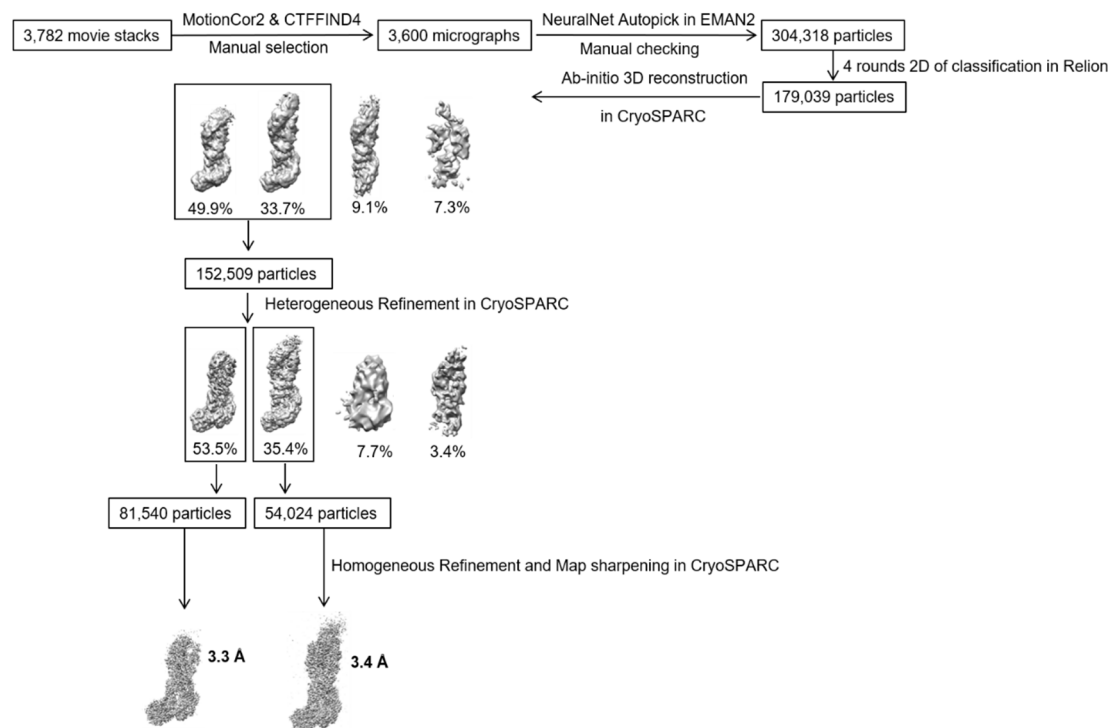

**Fig. S3** Workflow of data processing for apo Csm complexes.

Supplement: Supplementary file 3 — Supplementary information, Figure S3 [file 41422_2019_151_MOESM3_ESM.pdf]

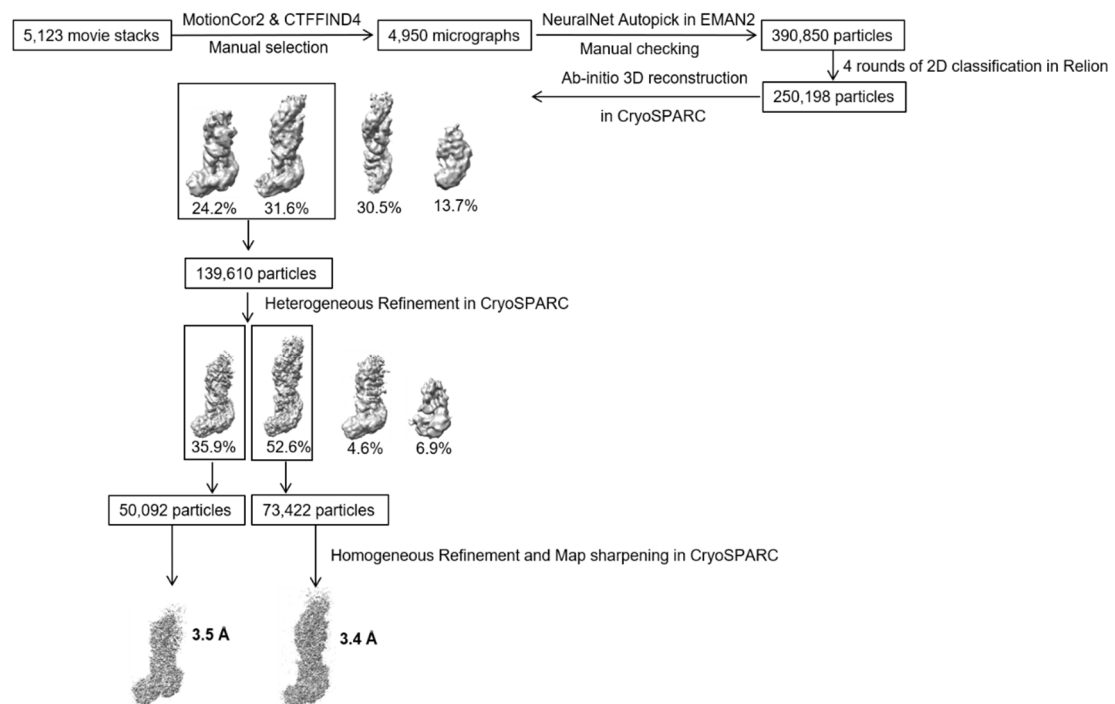

**Fig. S4** Workflow of data processing for target ssRNA-bound Csm complexes.

Supplement: Supplementary file 4 — Supplementary information, Figure S4 [file 41422_2019_151_MOESM4_ESM.pdf]
