# Supplementary material for: Coupling of ssRNA cleavage with DNase activity in type III-A CRISPR-Csm revealed by cryo-EM and biochemistry
Source: Cell Res. 2019 Feb 27;29(4):305–12. doi: 10.1038/s41422-019-0151-x (PMC6461802; doi:10.1038/s41422-019-0151-x)
Supplement: Supplementary file 5 — Supplementary information, Figure S5 [file 41422_2019_151_MOESM5_ESM.pdf]

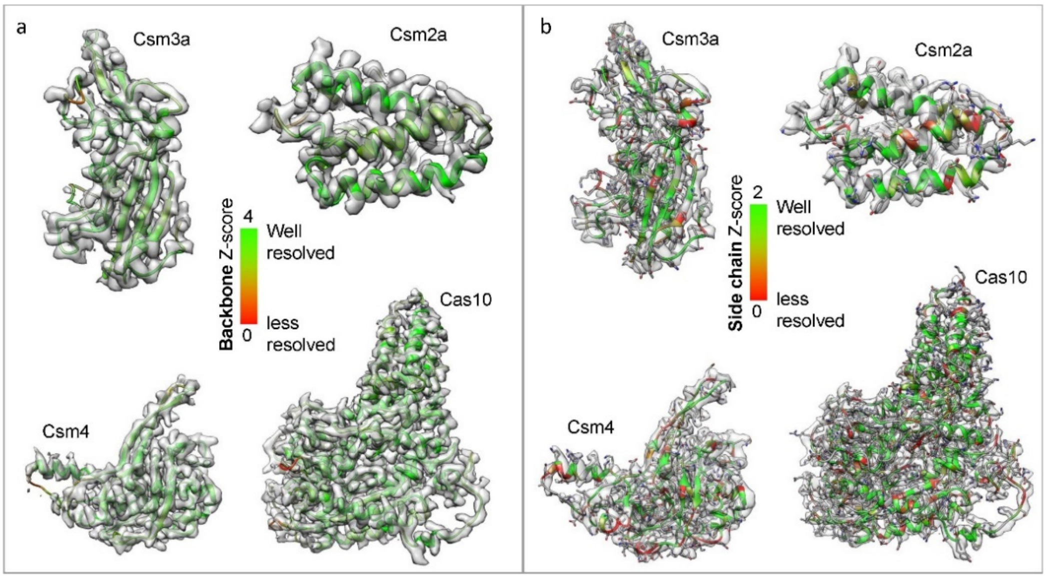

Fig. S5 Density maps for the primary components Cas10, Csm2a, Csm3a, and Csm4 with models. **a** The ribbon is color-coded by backbone Z-score. High Z-scores are seen for most parts of each model (green ribbon), though a few small loops are less resolved (red ribbon). **b** The ribbon is color-coded by side chain Z-score. Many side chains are well resolved (green), though some side chains, especially polar (e.g. ASP), are less resolved. Z-scores were not calculated for GLY and ALA residues (white ribbon).
