# Supplementary material for: Coupling of ssRNA cleavage with DNase activity in type III-A CRISPR-Csm revealed by cryo-EM and biochemistry
Source: Cell Res. 2019 Feb 27;29(4):305–12. doi: 10.1038/s41422-019-0151-x (PMC6461802; doi:10.1038/s41422-019-0151-x)
Supplement: Supplementary file 6 — Supplementary information, Figure S6 [file 41422_2019_151_MOESM6_ESM.pdf]

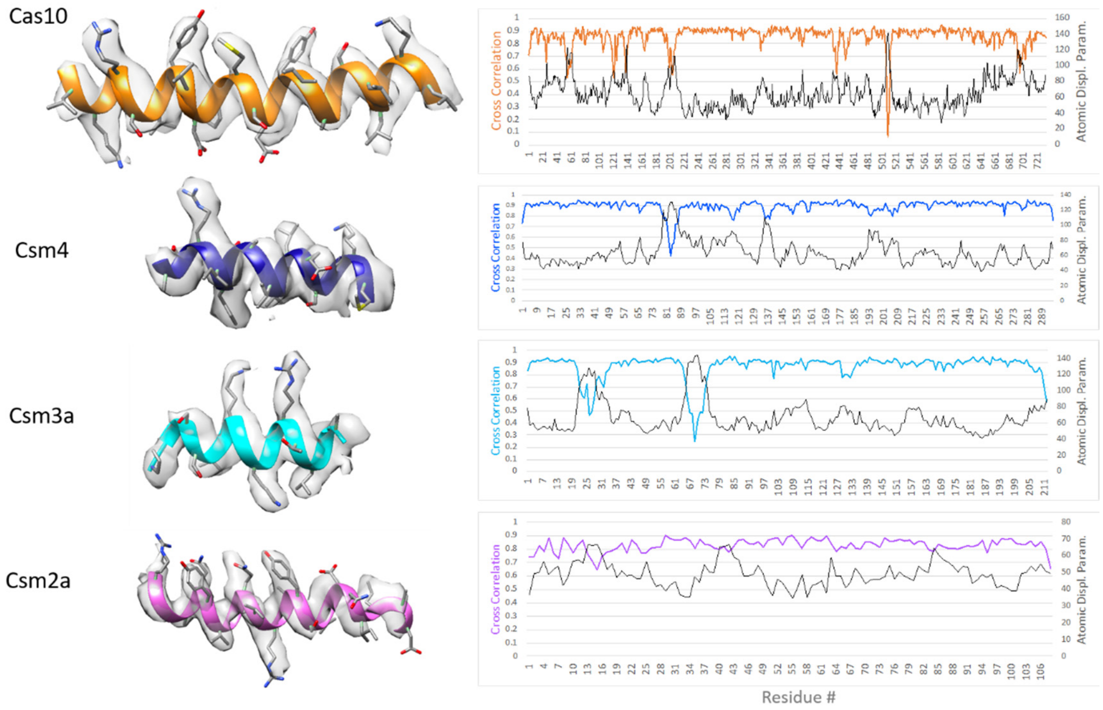

Fig. S6 Resolvability of the side chains in our cryo-EM structure. Left: representative helices from Cas10, Csm4, Csm3a and Csm2a. Right: Plotted scores for residues: per-residue cross-correlation coefficients (Cas10 - orange, Csm4 - blue, Csm3a - cyan, Csm2a - magenta), and per-residue atomic displacement parameter (black line).
