# Supplementary material for: Coupling of ssRNA cleavage with DNase activity in type III-A CRISPR-Csm revealed by cryo-EM and biochemistry
Source: Cell Res. 2019 Feb 27;29(4):305–12. doi: 10.1038/s41422-019-0151-x (PMC6461802; doi:10.1038/s41422-019-0151-x)
Supplement: Supplementary file 7 — Supplementary information, Figure S7 [file 41422_2019_151_MOESM7_ESM.pdf]

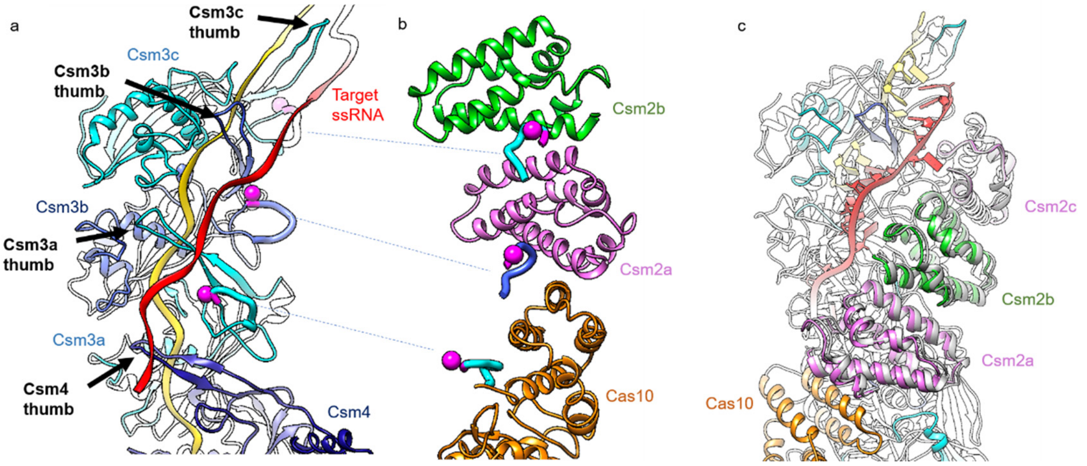

Fig. S7 Core region of the two spiral stacks. **a** Spiral stacking of Csm3 proteins. The thumbs of Csm4 and Csm3 are marked with black arrows. **b** Spiral stacking of Csm2 proteins. The loop of Csm3 containing the cleaving residue (Asp33, here mutated to Ala, purple sphere) is stabilized by Csm2. **c** Shift of Csm2 proteins from the apo state (gray ribbons) to target ssRNA-bound state (colored ribbons).
