# Supplementary material for: Coupling of ssRNA cleavage with DNase activity in type III-A CRISPR-Csm revealed by cryo-EM and biochemistry
Source: Cell Res. 2019 Feb 27;29(4):305–12. doi: 10.1038/s41422-019-0151-x (PMC6461802; doi:10.1038/s41422-019-0151-x)
Supplement: Supplementary file 8 — Supplementary information, Figure S8 [file 41422_2019_151_MOESM8_ESM.pdf]

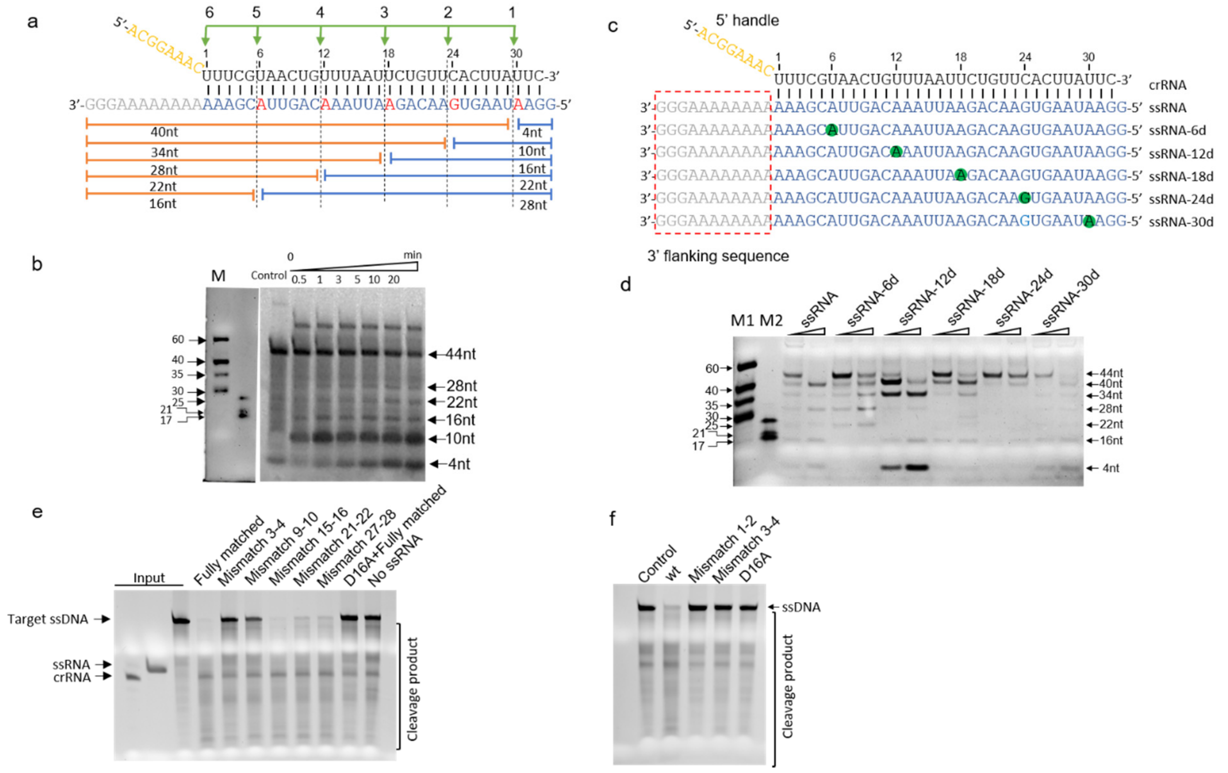

Fig. S8 The target ssRNA and the target ssDNA cleavage of Csm complex. **a** Sequences of crRNA and ssRNA. The nucleotide lengths of the observed cleavage products are indicated. **b** In vitro ssRNA cleavage assay using 5'-labled ssRNA. ssRNA substrates were 5'-end labeled with IRDye 800CW Maleimide. The samples were taken at the indicated time points (0.5, 1, 3, 5, 10, and 20 min). **c** Schematic representations of the 2'-deoxy-substituted RNAs. The 2'-deoxy-substituted bases are underlined in green. **d** Target ssRNA cleavage reactions with the 2'-deoxy-substituted RNAs as substrates, revealing the cleavage sites in the target RNAs. The reaction samples were taken at two time points of 4 min and 25 min. **e** Target ssDNA cleavage assay using 2-base pair mismatched ssRNA. **f** In vitro ssDNA cleavage assay using 2-base pair mismatched ssRNA.
