# Supplementary material for: Coupling of ssRNA cleavage with DNase activity in type III-A CRISPR-Csm revealed by cryo-EM and biochemistry
Source: Cell Res. 2019 Feb 27;29(4):305–12. doi: 10.1038/s41422-019-0151-x (PMC6461802; doi:10.1038/s41422-019-0151-x)
Supplement: Supplementary file 9 — Supplementary information, Figure S9 [file 41422_2019_151_MOESM9_ESM.pdf]

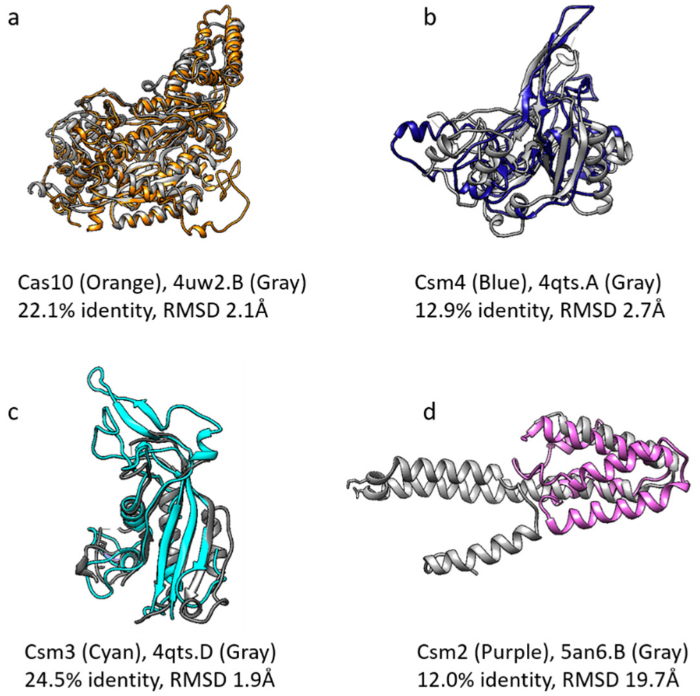

Fig. S9 Comparison with previous known Csm proteins in other species. The Csm proteins in our model are highlighted in orange (**a** Cas10), blue (**b** Cam4), cyan (**c** Csm3), and purple (**d** Csm2).
