# Supplementary material for: Coupling of ssRNA cleavage with DNase activity in type III-A CRISPR-Csm revealed by cryo-EM and biochemistry
Source: Cell Res. 2019 Feb 27;29(4):305–12. doi: 10.1038/s41422-019-0151-x (PMC6461802; doi:10.1038/s41422-019-0151-x)
Supplement: Supplementary file 15 — Supplementary Movie Legends [file 41422_2019_151_MOESM15_ESM.pdf]

Movies Legends

Movie S1 Overall structure (map and models) of the Type III-A CRISPR-Csm complex.

Movie S2 Zoom-in view of the ATP binding pocket in Cas10.

Movie S3 The base pairs between crRNA and target ssRNA are disrupted by the thumbs of Csm4 or Csm3 proteins at 6-nucleotide intervals.

Movie S4 The locations of the 3’-end of ssRNA and DNase active site.

Movie S5 Comparison with previous known Csm proteins in other species.
