# Supplementary material for: Coupling of ssRNA cleavage with DNase activity in type III-A CRISPR-Csm revealed by cryo-EM and biochemistry
Source: Cell Res. 2019 Feb 27;29(4):305–12. doi: 10.1038/s41422-019-0151-x (PMC6461802; doi:10.1038/s41422-019-0151-x)
Supplement: Supplementary file 16 — Supplementary information, Table S1 [file 41422_2019_151_MOESM16_ESM.pdf]

Supplementary information, Table S1 | **Data collection and refinement statistics**

	**Apo Csm**		**Target ssRNA-bound Csm**	
**Data collection and processing**				
Microscope	Titan Krios		Titan Krios	
Voltage (kV)	300		300	
Camera	Gatan K2 Summit		Gatan K2 Summit	
Pixel size (Å)	1.06		1.06	
Total Dose (e-/$Å^2$)	42.6		46.2	
Defocus range (μm)	0.7-2.7		0.7-2.5	
Number of micrographs	3,782		5,123	
Number of initial particles	304,318		390,850	
Symmetry	C1		C1	
Number of final particles	**Apo small**	**Apo big**	**Target small**	**Target big**
	81,540	84,024	50,092	73,422
Resolution (0.143 FSC, Å)	3.3	3.4	3.5	3.3
**Atomic model refinement**				
Software	Phenix 1.13 real-space-refine			
Number of protein residues	2412	3052	2423	3052
Number of atoms	19557	24707	20100	25311
Geometric parameters (r.m.s.d.)				
Bond length (Å)	0.008	0.007	0.006	0.009
Bond angel (°)	1.231	1.210	1.331	1.364
Ramachandran statistics				
Favored (%)	87.22	89.17	87.48	88.77
Allowed (%)	12.78	10.83	12.52	11.23
Disallowed (%)	0.00	0.00	0.00	0.00
Validation				
MolProbity score	1.94	1.87	1.98	2.01
Clashscore	5.92	5.51	6.69	5.39
Rotamer outliers (%)	0.94	0.86	0.85	1.53
