# Supplementary material for: Coupling of ssRNA cleavage with DNase activity in type III-A CRISPR-Csm revealed by cryo-EM and biochemistry
Source: Cell Res. 2019 Feb 27;29(4):305–12. doi: 10.1038/s41422-019-0151-x (PMC6461802; doi:10.1038/s41422-019-0151-x)
Supplement: Supplementary file 17 — Supplementary information, Table S2 [file 41422_2019_151_MOESM17_ESM.pdf]

Supplementary information, Table S2 | **Nucleic acid sequences used in the study**

**Nucleic sequence**	**Sequence (5' to 3')**
StCsm-pCRISPR_S3	GCCTTTATAGACCTTTAATCATATGGTACACTATAGATAGTGTTTCCAGTAGGTCCTACATCTTGTGCCTCTAGCAACTGCCTAGAGCACAAGATATGGGGATATAAACCTAATTACCTCGAGAGGGGACGGAAACTTTCGTAACTGTTTAATTCTGTTCACTTATTCCACCGATATAAACCTAATTACCTCGAGAGGGGACGGAAACTTTCGTAACTGTTTAATTCTGTTCACTTATTCCACCGATATAAACCTAATTACCTCGAGAGGGGACGGAAACTTTCGTAACTGTTTAATTCTGTTCACTTATTCCACCGATATAAACCTAATTACCTCGAGAGGGGACGGAAACTTTCGTAACTGTTTAATTCTGTTCACTTATTCCACCGATATAAACCTAATTACCTCGAGAGGGGACTTTTTT
40nt-crRNA	ACGGAAACUUUCGUAACUGUUUAAUUCUGUUCACUUAUUC
**ssRNA for cryo-EM**	
Target_ssRNA	GGGAAUAAGUGAACAGAAUUAAACAGUUACGAAAAAAAAAAAAGGGUACC
**RNA transcription assay DNA template**	
Target_ssRNA transcription template	ATGTAATACGACTCACTATAGGGAATAAGTGAACAGAATTAAACAGTTACGAAAAAAAAAAAAGGGTACC
**In vitro RNA cleavage assay**	
ssRNA-1	GGAAUAAGUGAACAGAAUUAAACAGUUACGAAACCACACCCGGG
Mismatch 1-2	GGAAUAAGUGAACAGAAUUAAACAGUUACGAUUCCACACCCGGG
Mismatch 3-4	GGAAUAAGUGAACAGAAUUAAACAGUUACCUAACCACACCCGGG
Mismatch 9-10	GGAAUAAGUGAACAGAAUUAAACUCUUACGAAACCACACCCGGG
Mismatch 15-16	GGAAUAAGUGAACAGAAAAAAACAGUUACGAAACCACACCCGGG
Mismatch 21-22	GGAAUAAGUGAUGAGAAUUAAACAGUUACGAAACCACACCCGGG
Mismatch 27-28	GGAAUUUGUGAACAGAAUUAAACAGUUACGAAACCACACCCGGG
ssRNA-2	GGAAUAAGUGAACAGAAUUAAACAGUUACGAAAAAAAAAAAAGGG
ssRNA-6d	GGAAUAAGUGAACAGAAUUAAACAGUU_dA_CGAAAAAAAAAAAAGGG
ssRNA-12d	GGAAUAAGUGAACAGAAUUAA_dA_CAGUUACGAAAAAAAAAAAAGGG
ssRNA-18d	GGAAUAAGUGAACAG_dA_AUUAAACAGUUACGAAAAAAAAAAAAGGG
ssRNA-24d	GGAAUAAGU_dG_AACAGAAUUAAACAGUUACGAAAAAAAAAAAAGGG
ssRNA-30d	GGA_dA_UAAGUGAACAGAAUUAAACAGUUACGAAAAAAAAAAAAGGG
Non_target_ssRNA	GGGUCACCUCCAAUGACUAGGGGUUUUAGAGCUAGAAAUAGCAAGUUAAAAUAAGGCUAGUCCGUUAUCAACUUGAAAAAGUGGCACCGAGUCGGUGCUU
**In vitro DNA cleavage assay**	
ssDNA-C	GAATAAGTGAACAGAATTAAACAGTTACGAAAAAAAGGCCGTAATATCCAGCTGAACGGTCTGGTATCAACGGTGGTATATCCAGTGA
ssDNA-NC	GTTCTTTACGATGCCATTGGGATAGGTCTTTAAAAAGGCCGTAATATCCAGCTGAACGGTCTGGTATCAACGGTGGTATATCCAGTGA
